# Reallocation of time between accelerometer-derived movement behaviors, genetic susceptibility, and risk of incident dementia, mortality, and premature death: a longitudinal cohort study

**DOI:** 10.1186/s12966-025-01814-8

**Published:** 2025-08-21

**Authors:** Wenya Zhang, Yang Pan, Yiwen Dai, Jie Liang, Jingya Ma, Yuling Liu, Darui Gao, Yanyu Zhang, Mengmeng Ji, Wuxiang Xie, Fanfan Zheng

**Affiliations:** 1 School of Nursing, Chinese Academy of Medical Sciences & Peking Union Medical College, 33 Ba Da Chu Road, Shijingshan District, Beijing, 100144 China; 2 Department of Endocrinology, Peking University First Hospital, No.8 Xishiku Road, Beijing, 100034 China; 3 Clinical Research Institute, Institute of Advanced Clinical Medicine, Peking University, No. 38 Xueyuan Road, Haidian District, Beijing, 100191 China; 4 Key Laboratory of Epidemiology of Major Diseases (Peking University), Ministry of Education, 38 Xueyuan Road, Haidian District, Beijing, 100191 China

**Keywords:** Time reallocation, Movement behaviors, Accelerometer, Dementia, Mortality

## Abstract

**Background:**

It is well established that all types of movement behaviors, including moderate-to-vigorous physical activity (MVPA), light-intensity physical activity (LIPA), sedentary behavior (SB), and sleep, are associated with the risk of incident dementia, all-cause mortality, and premature death. However, it remains unclear whether reallocating time from one type to another is associated with these outcomes. In addition, the extent to which genetic susceptibility modifies the association between physical activity and dementia risk still warrants further investigation.

**Methods:**

This study included 94 086 dementia-free participants from the UK Biobank with valid accelerometer and genomic data. Time spent MVPA, LIPA, SB, and sleep were derived from wrist-worn accelerometers. Genetic susceptibility of dementia was assessed by polygenic risk score (PRS) consisting of 82 single nucleotide polymorphisms. The isotemporal substitution model was applied to explore how reallocating time between movement behaviors was associated with incident dementia, mortality, and premature death.

**Results:**

Of 94 086 included participants, 52 853 (56.2%) were female, and the mean (standard deviation, SD) age was 62.3 (7.8) years. Reallocating 1 h/day to MVPA from LIPA, SB, and sleep was associated with a 19%, 26%, and 18% lower risk of incident dementia (adjusted hazard ratios [HRs] and 95% confidence intervals [CIs]: 0.81 [0.68, 0.95], 0.74 [0.63, 0.87], and 0.82 [0.69, 0.96], respectively). A 22%, 30%, and 29% reduced risk of mortality were observed when reallocating 1 h/day from LIPA, SB, and sleep to MVPA (0.78 [0.72, 0.84], 0.70 [0.65, 0.75], and 0.71 [0.66, 0.77], respectively). Replacing 1 h/day of SB with MVPA, LIPA, and sleep was associated with a 26%, 8%, and 9% lower risk of incident dementia (0.74 [0.63, 0.87], 0.92 [0.87, 0.97], and 0.91 [0.85, 0.97], respectively), and reallocating 1 h/day from SB to LIPA (0.89 [0.87–0.92]) or MVPA (0.70 [0.65–0.75]) was associated with reduced risk of mortality. Similar results could be seen in premature death. Participants with high levels of MVPA and low genetic risk showed 72% lower risk of dementia comparing to participants with low levels of MVPA and high PRS (0.28 [0.17–0.50]).

**Conclusions:**

Reallocating time to MVPA from any behavior and substituting physical activity of any intensity for SB were associated with decreased risks of incident dementia, mortality, and premature death, suggesting the significance of maintaining a physically active lifestyle among old adults. Moreover, increasing MVPA level could partially attenuate the strength of association between genetic susceptibility and the risk of dementia.

**Supplementary Information:**

The online version contains supplementary material available at 10.1186/s12966-025-01814-8.

## Background

Dementia remains a severe public health problem, with 57 million individuals worldwide living with dementia, and this staggering number is projected to escalate to 153 million by 2050 due to longevity and population aging [1]. Despite promising disease-modifying drugs for Alzheimer’s disease having been approved for the market in several countries, they have notable side effects, stringent eligibility, and high prices, thus limiting their extensive use in healthcare systems [2–4]. The 2024 update of the Lancet Commission on dementia prevention prioritizes tackling the 14 modifiable risk factors to delay the dementia onset, suggesting that being physically active could prevent 2% of dementia cases, and sleep was excluded as one of the risk factors due to inadequate consistent evidence [5]. However, the report was based on previous evidence that explored the independent associations of moderate-to-vigorous physical activity (MVPA), light-intensity physical activity (LIPA), sedentary behavior (SB), sleep, and risk of dementia, neglecting the intrinsic interplay and codependency of the four types of movement behaviors [6]. Because of the finite nature of a day, increasing time spent in one behavior must be compensated by decreasing time spent in others, which indicates the significance of unraveling the associations of time reallocation between different behaviors with the risk of incident dementia [7]. Moreover, most previous research adopted self-reported rather than accelerometer-derived data to measure the duration of movement behaviors, which may be subject to recall bias [8].

Genetic predisposition also contributes to the development of dementia, with evidence pinpointing the apolipoprotein E ε4 allele as an independent risk factor for Alzheimer’s disease [9]. Additionally, accumulating genome-wide association studies (GWAS) have recently revealed other genetic variants related to dementia risk, implicating that cumulative effects should be considered when identifying people of high dementia risk [10, 11]. Polygenic risk score (PRS) has been developed to quantify this effect, which has been validated as an ideal tool for risk stratification and disease prediction [12, 13]. Although there were former discussions about how dementia PRS interacted with lifestyle to modify cognitive function and subsequent risk of dementia; to our knowledge, the modifying role of genetic susceptibility on the relationship between MVPA levels and dementia risk has not been elucidated [14, 15]. Since high-intensity PA appeared to bring the most pronounced cognitive benefits [16], whether it could counteract the genetic predisposition associated with an increased risk of dementia remained to be explored.

Cohort studies investigating the relationships between different compositions of accelerometer-derived movement behaviors and mortality risk are emerging, but most of which employed self-reported sleep duration, had limited sample size, and failed to account for the associations of substitutions between movement behaviors (e.g., replacing 1 h per day from LIPA to MVPA on mortality risk) with the risk of mortality [17–20]. Recently, an isotemporal substitution model (ISM) has been developed for analyzing such data with a compositional attribute to address these concerns [21, 22], yet studies that illustrated the magnitude to which substituting one behavior with another lowers the risk of mortality and premature death were scarce. Furthermore, the majority of public health guidelines give physical activity (PA) recommendations based on their independent health benefits instead of a 24-hour activity spectrum approach [23–25], suggesting a need for better empirical evidence that integrates activities in the entire 24-hour day. 

The advantage of ISM in dealing with the compositional nature of data promotes its application in PA epidemiology, allowing the quantification of associations of time reallocation between movement behaviors and major health outcomes. Therefore, by using the accelerometer data from the UK Biobank (UKB), our study aimed to: (1) adopt ISM to examine the relationship between time reallocation of device-measured movement behaviors and the risk of incident dementia, mortality, and premature death; (2) explore whether the MVPA interacted with the genetic susceptibility quantified by PRS in relation to subsequent risk of dementia.

## Methods

### Study design and population

Our data came from the UKB upon request (project number 90492). The UKB is one of the largest public databases in the UK to date and encompasses detailed participant-level data on sociodemographic characteristics, health information, genetics, lifestyle habits, and medical records. Participants aged 40–49 years who registered in the UK’s National Health Service and lived within 25 miles of one of 22 assessment centers located throughout England, Wales, and Scotland were invited to participate in the baseline assessment. Ultimately, 502 411 participants consented to join this large cohort and attended one of the assessment centers during 2006–2010 for baseline assessments that involved a touchscreen questionnaire, verbal interview, physical examination, and bio-sample collection, resulting in a response rate of 5.4% [26]. Further information about UKB can be found on its official website (https://www.ukbiobank.ac.uk) and published articles [26–28]. Ethical consent was approved by the NHS North West Multi-center Research Ethics Committee (ethics approval number: 11/NW/0382, 16/NW/0274, and 21/NW/0157), and all participants provided informed consent before baseline data collection.

Between June 2013 and January 2016, UKB investigators initiated a sub-study to quantify the 24-hour movement behaviors of more than 100 000 participants via a wrist-worn accelerometer [29]. Individuals were asked to wear an Axivity AX3 triaxial accelerometer on the wrist of the dominant hand (Axivity, Newcastle, UK) for seven consecutive days as soon as they received it and mail the device back to the coordinating center at the end of the seven days. The raw accelerometer data were finally aggregated into 5-second epoch for summary data analysis [30]. A readable dataset was finally obtained from 103 579 participants, and the start time of wearing the device was considered the baseline of our study (Field ID: 90010).

**Fig. 1:**
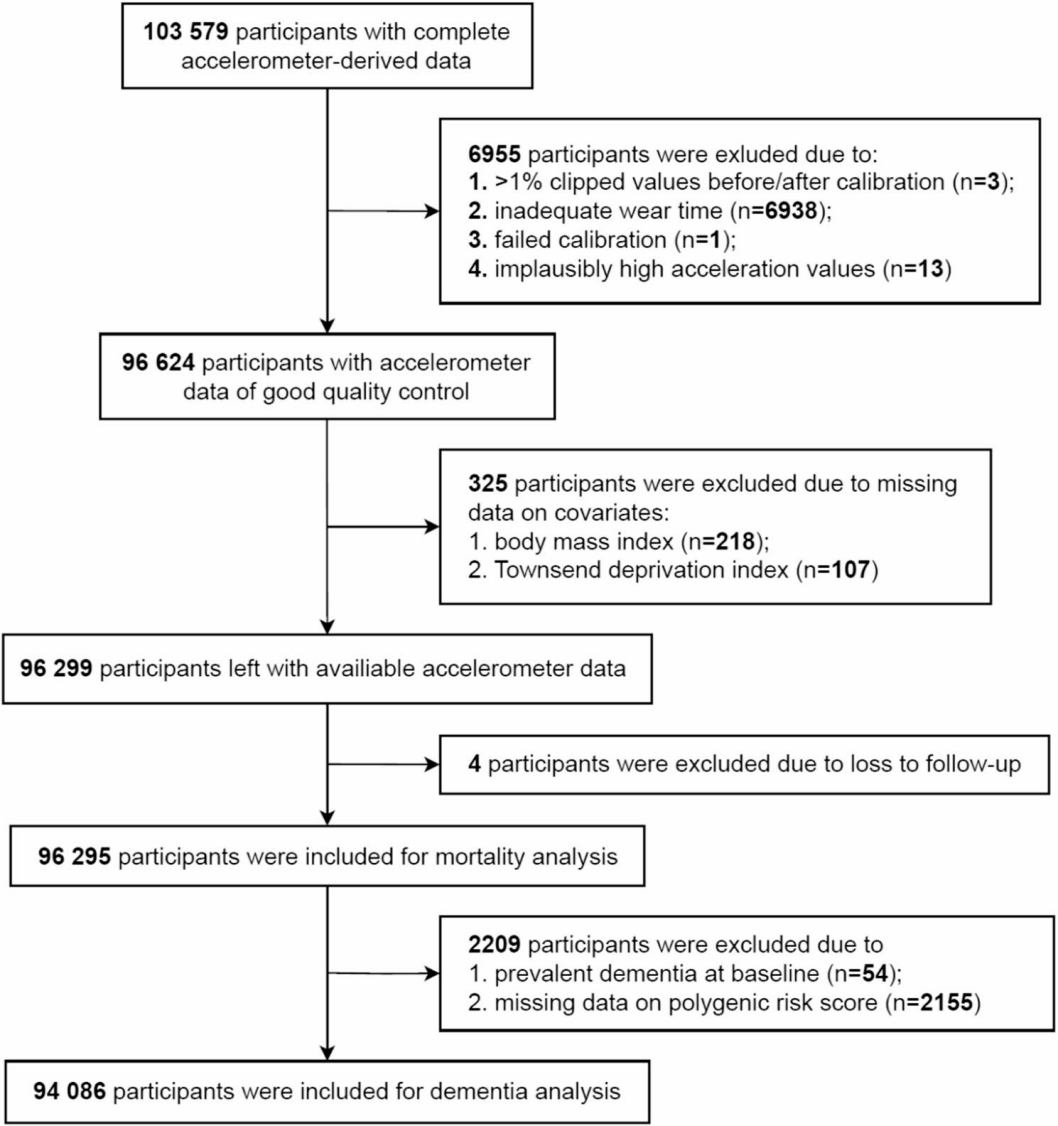
Flowchart of participants selection

Figure 1 pictures the process of selecting participants. According to established data processing rules [31], participants were excluded from further analyses due to invalid accelerometer data if the device failed to be calibrated (*n* = 1), if ‘clipped’ readings (exceeding the device’s sensor range of ± 8 gravities) were more than 1% of total readings before and after the calibration (*n* = 3), if wear time was inadequate (*n* = 6938), or if the acceleration average was implausibly high (> 100 milli-gravity, *n* = 13). Participants were further excluded due to loss to follow-up (*n* = 4) or missing covariates data (*n* = 325), leaving 96 295 individuals for mortality analyses. Participants with prevalent dementia at baseline (*n* = 54) and missing data on PRS (*n* = 2155) were further excluded for dementia analysis.

### Device-based measures of movement behaviors

As described above, movement behaviors were recorded via a wrist-worn triaxial accelerometer, including sleep, SB, LIPA, and MVPA, and they were classified based on a published machine-learning model [31]. In short, the model was trained on the accelerometer data from 152 British adults and then applied to classify the behavioral patterns of UKB participants [32]. Specifically, we used data in the UKB Data Showcase to quantify the average time spent in sleep, SB, LIPA, and MVPA per day (Field ID: 40046, 40047, 40048, and 40049).

### Outcomes

The outcomes of interest included dementia, mortality, and premature death. We utilized the algorithmically-defined outcomes to ascertain cases of all-cause dementia until Dec 31, 2022, which were algorithmic combinations of coded information from baseline self-reported medical condition codes, hospital records, and death registry data (Field ID: 42018, 42020, 42022, and 42024) [33]. Supplementary Table S1 lists the International Classification of Diseases Tenth Revision (ICD-10) codes used to define dementia, and the earliest recorded date, regardless of sources, was regarded as the dementia endpoint. The date of death was obtained through linkage to National Death Registries (Field ID: 40000) until Dec 31, 2022, and premature death was defined as death at age younger than 75 years old [34, 35].

### PRS for dementia

Whole-genome genotyping data in the UKB was assayed via the UK BiLEVE Axiom Array by Affymetrix (10% of participants) and the UK Biobank Axiom Array (90% of participants) that share 95% of marker content. Other detailed information regarding genetic data in the UKB was published elsewhere [36]. The PRS of all-cause dementia consisted of 82 single nucleotide polymorphisms (SNPs) according to a genome-wide association study (GWAS) conducted by the Mega Vascular Cognitive Impairment and Dementia (MEGAVCID) consortium [37]. We adopted the effect estimates of the 82 SNPs and their corresponding number of alleles in the UKB to calculate the weighted PRS based on the following formula:

$$\text{PRS}=\beta_{1}x_{1}+\beta_{2}x_{2}+\cdot\cdot\cdot\cdot+\beta_{\text{k}}x_{\text{k}}+\beta_{n}x_{n}$$

PRS = *β*_1_$$\:\mathcal{x}$$_1_ + *β*_2_$$\:\mathcal{x}$$_2_ + …. + *β*_k_$$\:\mathcal{x}$$_k_ + *β*_n_$$\:\mathcal{x}$$_n_

Where *β* represents the per-allele log odds ratio (OR) of the dementia-associated risk allele for SNP, $\:{x}_{k}$ is the number of alleles for the same SNP, and n means the total number of SNPs that were related to all-cause dementia. Supplementary Table S2 encloses detailed information regarding selected SNPs.

The PRS for all-cause dementia was further categorized into “low” (quartile 1), “intermediate” (quartile 2–3), and “high” (quartile 4) according to quartiles for statistical analyses, with a higher score indicating an elevated genetic susceptibility of dementia.

### Covariates

A comprehensive description of the covariates was provided in Supplementary Table S3. Covariates included age, sex, race (white or other ethnicity), educational attainment (higher educational level or not), smoking status (current smoking or not), alcohol intake (at least once per week or not), and depressed mood (yes or no). A higher educational level refers to attaining a college or university degree or other professional qualifications. Depressed mood was defined when the frequency of feeling down, depressed, or hopeless over the past 2 weeks was nearly every day or more than half the days. Body mass index (BMI) was calculated as weight in kilograms divided by height in meters squared. The Townsend deprivation index was calculated instantly prior to participants joining UKB based on the preceding national census output data. Hypertension was identified according to self-reported hypertension, use of antihypertensive medications, or physical measures (mean systolic blood pressure [SBP]/diastolic blood pressure [DBP] ≥ 140/90 mmHg). Likewise, diabetes was defined based on self-reported diabetes (diabetes, type 1 diabetes, or type 2 diabetes), use of antihyperglycemic drugs, or physical measures (plasma glycated hemoglobin [HbA1c] ≥ 48 mmol/mol or ≥ 6.5%). Coronary heart disease (CHD) and stroke were defined according to baseline self-reported data and verbal interviews with trained staff. Apolipoprotein E4 (APOE4) status (carrier, non-carrier) was based on genomic data [36]. Even though we preferred the follow-up data closest to the accelerometer study, the initial assessment data, spanning from 2006 to 2010, was ultimately adopted since roughly 70% of the covariates data were missing when using the follow-up data, and most covariates remained constant during the follow-up period.

### Statistical analysis

Comparisons of four movement behaviors based on different baseline characteristics were calculated via a Mann-Whitney U or Kruskal-Wallis test. To quantify the risks associated with reallocating 1 h per day from one behavior to another, we fitted the ISM as a basic Cox proportional hazards model with years between baseline to incident dementia, death, or follow-up years as the timescale [38, 39]. As described by published articles, all but one of the movement behaviors and covariates were simultaneously put into the model [21, 38, 39]. In our study, since participants were asked to wear the accelerometer the whole day, the sum of device-recorded time of sleep, SB, LIPA, and MVPA equals 24 h, which was not included in the ISM model [7]. The ISM expression can be briefly described as follows:

$$\begin{aligned} \mathrm{Dementia}\;\mathrm{risk}\:=&\:\mathrm{Intercept}\:+\:\mathrm b1\ast\mathrm{sleep}\:+\:\mathrm b2\ast\mathrm{SB}\:\\&+\:\mathrm b3\ast\mathrm{LIPA}\:+\:\mathrm b4\ast\mathrm{MVPA}\:\\&+\:\mathrm b5\ast\mathrm{covariates} \end{aligned}$$

By eliminating one behavior from the model, the coefficient of each behavior represents the estimated subsequent risk of developing dementia when substituting 1 h/day in the left-out behavior with 1 h/day in that behavior. For example, if sleep is removed from the model, b2 can be interpreted as the estimated risk of incident dementia when replacing 1 h/day of sleep with 1 h/day of SB. Comparable interpretations can be made for the remaining substitution models when omitting other behaviors (SB, LIPA, or MVPA) from the model. We employed identical models as mentioned above to analyze mortality and premature death risk. As we assumed that MVPA would bring the most prominent benefit for cognitive health, we further tested the interaction between MVPA levels and genetic risk on subsequent risk of dementia stratified by genetic susceptibility category, with participants of high PRS and low MVPA levels as reference. The definitions of “low”, “intermediate”, and “high” MVPA levels in the analysis were based on the tertiles of daily MVPA duration.

To verify the robustness of our results, we did several sensitivity analyses. First, the competing risk model was applied to consider death as a competing event when analyzing the association between replacement of movement behaviors and incident dementia, which is an optimal tool to evaluate competing actual risks [40]. Second, we excluded incident dementia or death cases within the first two years of follow-up to avoid reverse causality [41]. Third, because dementia prevalence increases with aging and is relatively low in younger participants, individuals younger than 50 years at baseline were excluded. Fourth, we divided the whole population into high or low SB duration group based on the tertiles and repeated the main analyses. Finally, subgroup analyses were performed to test whether age is a modifying factor in the associations between time reallocation and the risk of incident dementia, mortality, and premature death.

Statistical analyses were performed via SAS 9.4 (SAS Inst., Cary, NC, USA) and R 4.2.2 (R Foundation for Statistical Computing, Vienna, Austria). All analyses were two-sided, and the statistical significance was set at *p* < 0.05.

## Results

### Baseline characteristics

Supplementary Table S4 displays the movement behaviors of 94 086 participants by baseline characteristics. The median composition of movement behaviors in the population was 8.7 h/day sleep, 9.4 h/day SB, 4.9 h/day LIPA, and 33 min/day MVPA. Younger participants are more active in doing MVPA than senior participants. Females spend significantly more time in sleep and LIPA than males, and less time in SB and MVPA. Individuals with higher BMI tend to have higher levels of sedentary time and lower levels of LIPA and MVPA.

### Associations with incident dementia

Over a median follow-up of 8.1 years (interquartile range [IQR]: 7.6–8.6 years), 788 dementia cases were ascertained. Table 1 displays the hazard ratios for incident dementia associated with reallocating time between different movement behaviors in detail. Substituting sedentary time with other behaviors was associated with a decreased risk of developing dementia. In specific, replacing 1 h/day sedentary time with LIPA was associated with an 8% lower risk of subsequent dementia (adjusted HR, 0.92; 95% CI: 0.87–0.97, *P* = 0.004), while reallocating 1 h/day from LIPA to sedentary time was associated with a 9% higher risk of developing dementia (adjusted HR, 1.09; 95% CI: 1.03–1.15, *P* = 0.004). Notably, substituting MVPA for other behaviors was associated with a more prominent lower risk of dementia. In contrast, the reallocation between time spent in sleep and LIPA was not associated with the risk of subsequent dementia.

**Table 1:** Hazard ratios for incident dementia estimated using a multivariable-adjusted isotemporal substitution Cox regression model among 94 086 participants

	Sleep	SB	LIPA	MVPA
Model 1^a^				
Replace sleep with	Replaced	1.06 (0.99, 1.14)	0.93 (0.87, 1.01)	0.64 (0.54, 0.75)^*^
Replace SB with	0.94 (0.88, 1.01)	Replaced	0.88 (0.83, 0.93)^*^	0.60 (0.51, 0.71)^*^
Replace LIPA with	1.07 (0.99, 1.15)	1.14 (1.08, 1.20)^*^	Replaced	0.69 (0.58, 0.81)^*^
Replace MVPA with	1.56 (1.33, 1.84)^*^	1.66 (1.42, 1.95)^*^	1.46 (1.24, 1.72)^*^	Replaced
Model 2^b^				
Replace sleep with	Replaced	1.10 (1.03, 1.17)	1.01 (0.93, 1.08)	0.78 (0.66, 0.92)
Replace SB with	0.91 (0.85, 0.98)	Replaced	0.92 (0.87, 0.97)	0.71 (0.61, 0.83)^*^
Replace LIPA with	0.99 (0.92, 1.07)	1.09 (1.03, 1.15)	Replaced	0.77 (0.66, 0.91)
Replace MVPA with	1.28 (1.09, 1.51)	1.41 (1.21, 1.64)^*^	1.29 (1.10, 1.52)	Replaced
Model 3^c^				
Replace sleep with	Replaced	1.10 (1.03, 1.17)	1.01 (0.94, 1.09)	0.82 (0.69, 0.96)
Replace SB with	0.91 (0.85, 0.97)	Replaced	0.92 (0.87, 0.97)	0.74 (0.63, 0.87)^*^
Replace LIPA with	0.99 (0.92, 1.06)	1.09 (1.03, 1.15)	Replaced	0.81 (0.68, 0.95)
Replace MVPA with	1.23 (1.04, 1.45)	1.35 (1.16, 1.58)^*^	1.24 (1.05, 1.47)	Replaced
*LIPA* light-intensity physical activity, *MVPA *moderate-to-vigorous physical activity, *SB* sedentary behavior. One asterisk (*) indicates *P-*value < 0.001.				

^a^ No covariates were adjusted in the model.

^b^ Adjusted covariates include age, sex, race, education, and Townsend index.

^c^ Adjusted all covariates in Model 2 and body mass index, current smoking, current alcohol consumption, depressed mood, hypertension, diabetes, coronary heart disease, stroke, and apolipoprotein E4 status.

### Associations with mortality and premature death

4117 death cases were identified during a median follow-up of 8.1 years (IQR: 7.6–8.6 years). As demonstrated in Table 2, replacing LIPA with sleep (adjusted HR, 1.10; 95% CI: 1.07–1.13, *P* < 0.001) or SB (adjusted HR, 1.12; 95% CI: 1.09–1.15, *P* < 0.001) was associated with higher mortality risk, while replacing LIPA with MVPA was associated with lower mortality risk (adjusted HR, 0.78; 95% CI: 0.72–0.84, *P* < 0.001). Likewise, reallocating other behaviors to MVPA was associated with a more distinguished lower mortality risk. However, the reallocation of time between sleep and SB was not associated with mortality risk. Similar results were observed for premature death (Table 3).

**Table 2:** Hazard ratios for mortality estimated using a multivariable-adjusted isotemporal substitution Cox regression model among 96 295 participants

	Sleep	SB	LIPA	MVPA
Model 1^a^				
Replace sleep with	Replaced	1.01 (0.98, 1.03)	0.82 (0.80, 0.85)^*^	0.59 (0.55, 0.64)^*^
Replace SB with	1.00 (0.97, 1.02)	Replaced	0.82 (0.80, 0.84)^*^	0.59 (0.54, 0.63)^*^
Replace LIPA with	1.22 (1.18, 1.26)^*^	1.22 (1.20, 1.25)^*^	Replaced	0.72 (0.67, 0.78)^*^
Replace MVPA with	1.70 (1.57, 1.83)^*^	1.70 (1.58, 1.84)^*^	1.39 (1.29, 1.50)^*^	Replaced
Model 2^b^				
Replace sleep with	Replaced	1.02 (1.00, 1.05)	0.89 (0.86, 0.92)^*^	0.62 (0.58, 0.67)^*^
Replace SB with	0.98 (0.95, 1.01)	Replaced	0.87 (0.85, 0.89)^*^	0.61 (0.57, 0.66)^*^
Replace LIPA with	1.13 (1.09, 1.16)^*^	1.15 (1.12, 1.18)^*^	Replaced	0.70 (0.65, 0.76)^*^
Replace MVPA with	1.61 (1.49, 1.74)^*^	1.64 (1.52, 1.77)^*^	1.43 (1.32, 1.54)^*^	Replaced
Model 3^c^				
Replace sleep with	Replaced	1.02 (0.99, 1.04)	0.91 (0.88, 0.94)^*^	0.71 (0.66, 0.77)^*^
Replace SB with	0.98 (0.96, 1.01)	Replaced	0.89 (0.87, 0.92)^*^	0.70 (0.65, 0.75)^*^
Replace LIPA with	1.10 (1.07, 1.13)^*^	1.12 (1.09, 1.15)^*^	Replaced	0.78 (0.72, 0.84)^*^
Replace MVPA with	1.41 (1.31, 1.52)^*^	1.44 (1.33, 1.54)^*^	1.28 (1.19, 1.38)^*^	Replaced

*LIPA *light-intensity physical activity, MVPA moderate-to-vigorous physical activity*, SB *sedentary behavior. One asterisk (*) indicates* P-*value < 0.001.

^a^No covariates were adjusted in the model.

^b^Adjusted covariates include age, sex, race, education, and Townsend index.

^c^Adjusted all covariates in Model 2 and body mass index, current smoking, current alcohol consumption, depressed mood, hypertension, diabetes, coronary heart disease, stroke, and apolipoprotein E4 status.

**Table 3:** Hazard ratios for premature death estimated using a multivariable-adjusted isotemporal substitution Cox regression model among 96 295 participants

	Sleep	SB	LIPA	MVPA
Model 1^a^				
Replace sleep with	Replaced	1.01 (0.98, 1.04)	0.82 (0.80, 0.85)^*^	0.60 (0.56, 0.65)^*^
Replace SB with	0.99 (0.96, 1.02)	Replaced	0.82 (0.80, 0.84)^*^	0.60 (0.55, 0.64)^*^
Replace LIPA with	1.21 (1.18, 1.25)^*^	1.23 (1.20, 1.26)^*^	Replaced	0.73 (0.67, 0.79)^*^
Replace MVPA with	1.66 (1.54, 1.80)^*^	1.68 (1.56, 1.82)^*^	1.37 (1.27, 1.49)^*^	Replaced
Model 2^b^				
Replace sleep with	Replaced	1.03 (1.00, 1.05)	0.89 (0.86, 0.92)^*^	0.63 (0.58, 0.68)^*^
Replace SB with	0.98 (0.95, 1.00)	Replaced	0.87 (0.84, 0.89)^*^	0.61 (0.57, 0.66)^*^
Replace LIPA with	1.13 (1.09, 1.16)^*^	1.16 (1.13, 1.19)^*^	Replaced	0.71 (0.65, 0.77)^*^
Replace MVPA with	1.60 (1.47, 1.73)^*^	1.64 (1.51, 1.77)^*^	1.42 (1.30, 1.54)^*^	Replaced
Model 3^c^				
Replace sleep with	Replaced	1.02 (0.99, 1.05)	0.91 (0.88, 0.94)^*^	0.72 (0.66, 0.77)^*^
Replace SB with	0.98 (0.95, 1.01)	Replaced	0.89 (0.87, 0.91)^*^	0.70 (0.65, 0.76)^*^
Replace LIPA with	1.10 (1.07, 1.14)^*^	1.12 (1.10, 1.15)^*^	Replaced	0.79 (0.73, 0.85)^*^
Replace MVPA with	1.40 (1.29, 1.51)^*^	1.43 (1.32, 1.54)^*^	1.27 (1.17, 1.38)^*^	Replaced

*LIPA* light-intensity physical activity, MVPA moderate-to-vigorous physical activity, SB sedentary behavior. One asterisk (*) indicates* P*-value < 0.001.

^a^No covariates were adjusted in the model.

^b^Adjusted covariates include age, sex, race, education, and Townsend index.

^c^Adjusted all covariates in Model 2 and body mass index, current smoking, current alcohol consumption, depressed mood, hypertension, diabetes, coronary heart disease, stroke, and apolipoprotein E4 status.

### The modifying role of genetic susceptibility

**Fig. 2:**
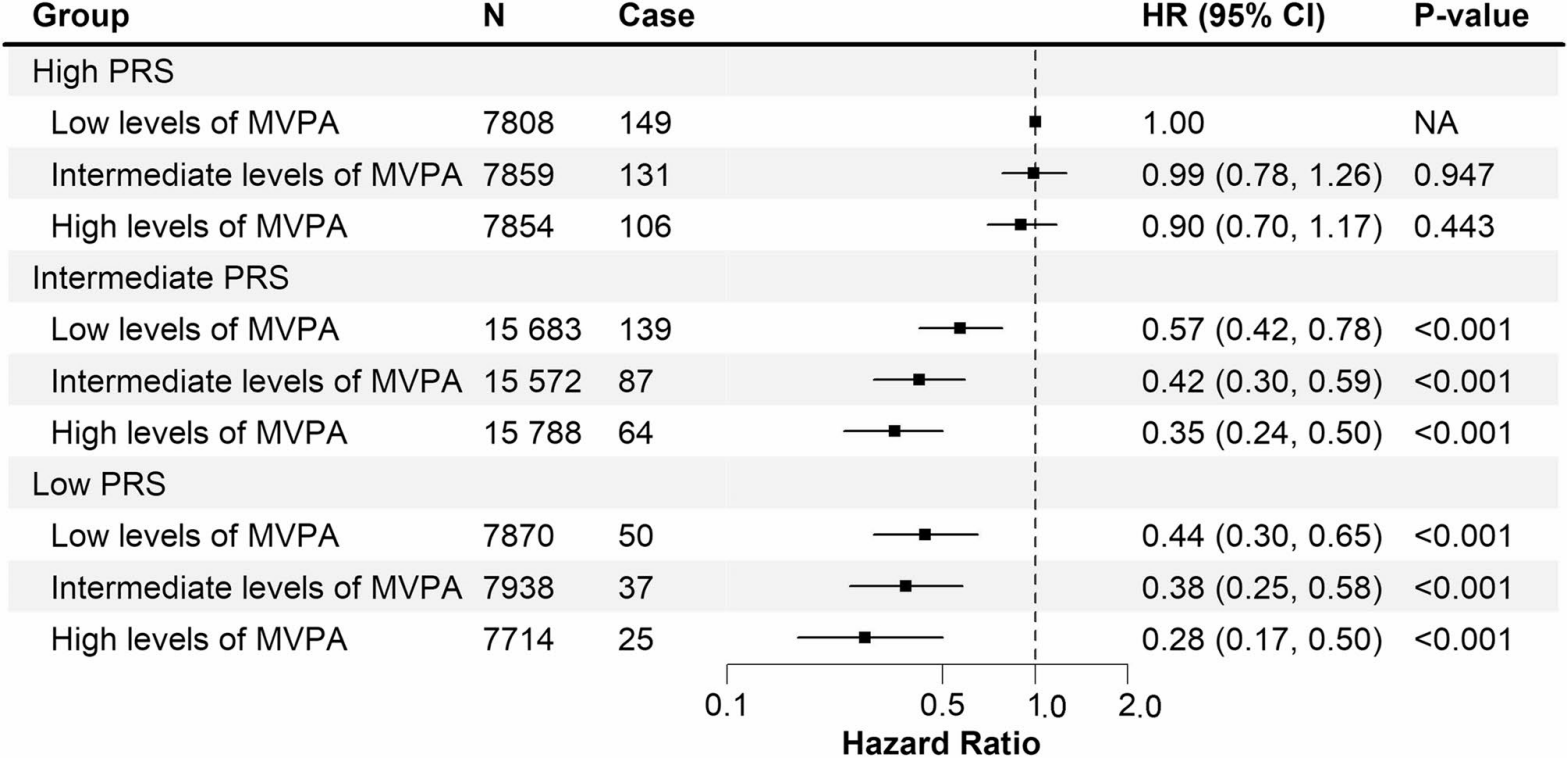
The joint association of MVPA levels and genetic susceptibility on the risk of incident dementia (*n* = 94 086)

Compared with individuals in high PRS and low levels of MVPA, the lowest risk of dementia was observed among individuals with a low PRS and high levels of MVPA (adjusted HR, 0.28; 95% CI: 0.17–0.50, *P* < 0.001) (Fig. 2). Moreover, the HRs decreased with the increase of MVPA levels within the low and intermediate PRS categories. Interestingly, the HRs of the intermediate and high MVPA group in the intermediate PRS category were comparable to the HRs of the low and intermediate MVPA group in the low PRS category, respectively.

### Sensitivity analysis

Compared with the results of the main analyses, the results of the sensitivity analyses remained stable (Supplementary Tables S5-8). For example, significant associations of reallocating time to MVPA with reduced risk of incident dementia were still observed, except that the strengths of associations were slightly elevated in competing risk models (Supplementary Table S5). Similar results were found when excluding dementia cases within 2 years since baseline or participants aged younger than 50 years at baseline (Supplementary Tables S6 and S8).

## Discussion

Our research investigated the associations between reallocation of time across four types of 24-hour activities recorded by wrist-worn accelerometers and risk of incident dementia, mortality, and premature death by using ISM. The results illustrated that replacing 1 h/day of other behaviors with an equal amount of time spent in MVPA was associated with an 18–26% lower risk of dementia, a 22–30% reduced risk of mortality, and a 21–30% decreased risk of premature death. Reallocating 1 h/day of SB to other behaviors was associated with an 8–26% lower risk of dementia, and substituting 1 h/day of SB with LIPA or MVPA was associated with an 11–30% decreased risk of mortality as well as premature death. Additionally, individuals with a low PRS and intermediate level of MVPA have a similar risk of developing dementia compared to individuals with an intermediate PRS and high MVPA level, suggesting the elevated risk associated with genetic predisposition to dementia could be partly offset by increasing MVPA level. However, the reallocation of time between LIPA and sleep was not associated with dementia risk in the multivariable-adjusted model, and the reallocation of time spent in SB and sleep was not associated with subsequent risk of mortality or premature death.

Presently, recall bias and reverse causality were likely to exist in most relevant studies due to the use of self-reported data and cross-sectional designs [42, 43]. Meanwhile, most research employed thresholds of PA and sleep to group participants and then compared the dementia risk across groups, which failed to account for the inherent interconnection of movement behaviors [44]. Despite these, our findings were consistent with prior studies, suggesting that higher-intensity PA may exert a more prominent benefit on cognitive function [42–45]. For example, results from the Coronary Artery Risk Development in Young Adults (CARDIA) study showed that replacing 30 min of LIPA with an equal amount of time spent in MVPA was associated with higher cognitive function scores [45]. Notably, we also found that substituting time spent in SB with other behaviors was associated with a reduced risk of dementia, which reaffirmed the slogan ‘every move counts’ promoted by the World Health Organization [24]. Interestingly, the reallocation of time between LIPA and sleep was not associated with dementia risk in our study, indicating that sleep might bring comparable positive benefits on cognitive function as LIPA through multiple distinct mechanisms, such as clearance of neurotoxins [46], reduction of systemic inflammation [47], and maintenance of neural hemostasis [48].

Furthermore, we observed that the high genetic susceptibility of dementia could be partially offset by increasing MVPA levels for the first time, with greater benefits associated with higher MVPA levels within each genetic risk category, except for participants in the high PRS group. It is worthwhile to mention that several previous studies reported the protective benefit of single or multiple lifestyle factors on dementia risk irrespective of genetic susceptibility [15, 49, 50], while this beneficial role disappeared among high genetic risk groups for populations aged older than 60 years [51, 52]. Our findings added evidence to this topic, indicating that the cumulation of neuronal damages dominated by genetic predisposition due to aging ultimately plays a deciding role in the development of dementia for older adults [52, 53]. On the optimistic side, 75% of the population in our study, namely those at intermediate and low genetic risk, could potentially alleviate their dementia risk up to 72% if adhering to a physically active lifestyle.

Another important finding of our study was that substituting time from other behaviors with MVPA provided the greatest benefit against mortality and premature death, which is consistent with previous studies [54–56]. Results of an individual participant meta-analysis, leveraging accelerometer-derived data from six cohorts, demonstrated that a relatively higher proportion of MVPA over 24 h is significantly associated with the lowest mortality risk [56]. Moreover, reallocating more time from sleep or SB to LIPA exerted a positive benefit against mortality and premature death, which has paramount public health relevance since increasing the time of LIPA, such as mopping, washing dishes, and walking, was much easier for people to accomplish [55]. More importantly, engaging in PA with any intensity rather than sitting was associated with an 11–30% lower risk of mortality and premature death, which poses the necessity to interrupt the continuity of sitting [57]. However, we observed that reallocating time between sleep and SB was not associated with mortality and premature death, which may imply the limited benefit of sleep on the longevity of the population.

The major merit of our study was the application of ISM in a prospective cohort with a large sample, which considered the interdependency of movement behaviors and presented quantitative estimates of the risks of multiple health outcomes. Several former studies constructed isometric log ratios to conduct compositional analysis rather than ISM when exploring the relationships between time reallocation and health outcomes [31, 43, 54–56]; however, considering that current PA guidelines are conveyed in absolute numbers when making recommendations and that identical outcomes could be obtained using either model, the interpretation of ISM is more intuitive and easier to comprehend with comparable accuracy [22]. Second, movement behaviors were estimated via wearable devices and categorized based on an established machine-learning model [31], which minimized the possibility of recall bias. Third, unlike former studies using PRS of Alzheimer’s disease to represent the genetic risk of all-cause dementia, the PRS of all-cause dementia in our study was calculated based on the most updated GWAS, which accounted for more putative genetic loci of dementia [37]. Fourth, we quantified the modification role of genetic susceptibility on the association between a specific type of PA and dementia risk for the first time, reaffirming the prominent role of MVPA on protecting cognitive health.

Our study also has several limitations. A primary limitation of our research was the inability to explore cause-effects because of the observational design. Second, we merely considered sleep duration due to limited data, which might bias the observed associations since other sleep traits, such as sleep apnea, chronotype, and insomnia, were proven to be associated with the risk of dementia [58]. Third, since the absolute values of sleep, SB, LIPA, and MVPA durations were used, the ISM failed to adequately account for the collinearity between the four types of movement behaviors, which might have biased the findings toward the null. Fourth, the sample overlap between MEGAVCID consortium and our study might inflate the association between the PRS and dementia risk, suggesting the need for future validation of PRS in another independent dataset. Fifth, residual confounders cannot be ruled out even though many acknowledged confounders were adjusted in the model. Sixth, due to the significant differences in baseline characteristics between participants included and excluded (Supplementary Table S15), excluding 9493 participants might result in selection bias. Seventh, 96.6% of volunteers in our sample were white ethnicity background, which hampered the generalizability of results. Large-scale population-based cohort studies involving individuals of non-white ancestry are warranted to provide unbiased statistics in multiethnic populations and to facilitate further comparison of the findings.

## Conclusions

In summary, by utilizing accelerometer data in the UKB, we first restated the necessity of increasing MVPA and substituting SB with light to vigorous PA whenever possible to maintain cognitive health and achieve longevity, and then found that elevating MVPA levels could partially counteract the higher risk associated with genetic susceptibility to dementia.

## Supplementary Information

Supplementary Material 1.

## Data Availability

No datasets were generated or analysed during the current study.

## Abbreviations

APOE4: Apolipoprotien E4
BMI: Body mass index
CHD: Coronary heart disease
CI: Confidence interval
DBP: Diastolic blood pressure
GWAS: Genome-wide association studies
HbA1c: Glycated hemoglobin
HR: Hazard ratio
ICD-10: International classification of diseases tenth revision
IQR: Interquartile range
ISM: Isotemporal substitution model
LIPA: Light-intensity physical activity
MEGAVCID: Mega vascular cognitive impairment and dementia
MVPA: Moderate-to-vigorous physical activity
OR: odds ratio
PA: Physical activity
