# Supplementary material for: Reallocation of time between accelerometer-derived movement behaviors, genetic susceptibility, and risk of incident dementia, mortality, and premature death: a longitudinal cohort study
Source: Int J Behav Nutr Phys Act. 2025 Aug 21;22:112. doi: 10.1186/s12966-025-01814-8 (PMC12369132; doi:10.1186/s12966-025-01814-8)
Supplement: Supplementary file 1 — Supplementary Material 1. [file 12966_2025_1814_MOESM1_ESM.docx]

**Supplemental Online Content**

**Table S1** The International Classification of Diseases Tenth Revision (ICD-10) codes used to ascertain all-cause dementia cases

**Table S2** Detailed information of SNPs used to construct the polygenic risk score (PRS) for all-cause dementia

**Table S3** Definition and assessment of covariates

**Table S4** Movement Behaviors of 94 086 Study Participants by Baseline Characteristics

**Table S5** Associations of reallocation of time between different types of movement behaviors and incident dementia: competing risk model (n=94 086)

**Table S6** Associations of reallocation of time between different types of movement behaviors and incident dementia after excluding dementia diagnosed within 2 years since baseline (n=94 037)

**Table S7** Associations of reallocation of time between different types of movement behaviors and mortality after excluding death within 2 years since baseline (n=95 748)

**Table S8** Associations of reallocation of time between different types of movement behaviors and incident dementia after excluding participants aged <50 years at baseline (n=87 296)

**Table S9** Associations of reallocation of time between different types of movement behaviors and incident dementia among participants with high or low duration of sedentary behavior (n=94 086)

**Table S10** Associations of reallocation of time between different types of movement behaviors and mortality among participants with high or low duration of sedentary behavior (n=96 295)

**Table S11** Associations of reallocation of time between different types of movement behaviors and premature death among participants with high or low duration of sedentary behavior (n=96 295)

**Table S12** Associations of reallocation of time between different types of movement behaviors and dementia, stratified by baseline age (n=94 086)

**Table S13** Associations of reallocation of time between different types of movement behaviors and mortality, stratified by baseline age (n=96 295)

**Table S14** Associations of reallocation of time between different types of movement behaviors and premature death, stratified by baseline age (n=96 295)

**Table S15** Comparison between baseline characteristics between participants included (n=94 086) and excluded (n=9493) due to invalid accelerometer and genomic data, history of dementia, or missing data of covariates

**Table S1** The International Classification of Diseases Tenth Revision (ICD-10) codes used to ascertain all-cause dementia cases

| **ICD 10 Code** | **Code description** | **All-cause dementia** |
| --- | --- | --- |
| A81.0 | Sporadic Creutzfeldt-Jakob disease | 1 |
| F00 | Dementia in Alzheimer's disease | 1 |
| F00.0 | Dementia in Alzheimer's disease with early onset | 1 |
| F00.1 | Dementia in Alzheimer's disease with late onset | 1 |
| F00.2 | Dementia in Alzheimer's disease, atypical or mixed type | 1 |
| F00.9 | Dementia in Alzheimer's disease, unspecified | 1 |
| F01 | Vascular dementia | 1 |
| F01.0 | Vascular dementia of acute onset | 1 |
| F01.1 | Multi-infarct dementia | 1 |
| F01.2 | Subcortical vascular dementia | 1 |
| F01.3 | Mixed cortical and sub-cortical vascular dementia | 1 |
| F01.8 | Other vascular dementia | 1 |
| F01.9 | Vascular dementia, unspecified | 1 |
| F02 | Dementia in other diseases classified elsewhere | 1 |
| F02.0 | Dementia in Picks disease | 1 |
| F02.1 | Dementia in Creutzfeldt-Jacob disease | 1 |
| F02.2 | Dementia in Huntington’s disease | 1 |
| F02.3 | Dementia in Parkinson’s disease | 1 |
| F02.4 | Dementia in HIV disease | 1 |
| F02.8 | Dementia in other specified diseases classified elsewhere | 1 |
| F03 | Unspecified dementia | 1 |
| F05.1 | Delirium superimposed on dementia | 1 |
| F10.6 | Mental and behavioral disorders due to use of alcohol - amnesic syndrome | 1 |
| G30 | Alzheimer’s disease | 1 |
| G30.0 | Alzheimer’s disease with early onset | 1 |
| G30.1 | Alzheimer’s disease with late onset | 1 |
| G30.8 | Other Alzheimer's disease | 1 |
| G30.9 | Alzheimer's disease unspecified | 1 |
| G31.0 | Circumscribed brain atrophy | 1 |
| G31.1 | Senile degeneration of brain | 1 |
| G31.8 | Other specified degenerative diseases of nervous system | 1 |
| I67.3 | Binswanger's disease | 1 |

1=yes, 0=no.

**Table S2** Detailed information of SNPs used to construct the polygenic risk score (PRS) for all-cause dementia

| **SNP** | **Chromosome** | **Position** | **Effect allele** | **Beta^a^** |
| --- | --- | --- | --- | --- |
| rs429358 | 19 | 45411941 | C | 1.1728 |
| rs4663105 | 2 | 127891427 | C | 0.156 |
| rs12453 | 11 | 59945745 | C | -0.0933 |
| rs10792832 | 11 | 85867875 | A | -0.0857 |
| rs10948367 | 6 | 47585615 | G | 0.0886 |
| rs4295 | 17 | 61556298 | C | -0.0784 |
| rs4844610 | 1 | 207802552 | A | 0.0961 |
| rs2906644 | 7 | 99956290 | G | -0.1016 |
| rs3764650 | 19 | 1046520 | G | 0.1141 |
| rs9323877 | 14 | 92934269 | G | 0.0699 |
| rs1532278 | 8 | 27466315 | T | -0.0629 |
| rs7912495 | 10 | 11718713 | G | 0.0684 |
| rs17125924 | 14 | 53391680 | G | 0.1009 |
| rs11767557 | 7 | 143109139 | C | -0.0742 |
| rs1854554 | 9 | 92155871 | A | 0.0598 |
| rs7118826 | 11 | 26195535 | G | 0.0686 |
| rs79832570 | 8 | 145097720 | C | 0.1433 |
| rs13010870 | 2 | 151765163 | C | -0.0732 |
| rs1354106 | 19 | 51737991 | G | -0.0614 |
| rs6014724 | 20 | 54998544 | G | -0.1071 |
| rs897150 | 8 | 126576702 | A | -0.0634 |
| rs11168036 | 5 | 139707439 | T | 0.0572 |
| rs17269688 | 2 | 106469267 | G | -0.1937 |
| rs677649 | 7 | 123439244 | T | 0.0851 |
| rs8081878 | 17 | 47436812 | T | 0.057 |
| rs4654450 | 1 | 4667378 | G | -0.0697 |
| rs11218343 | 11 | 121435587 | C | -0.1658 |
| rs2297508 | 17 | 17715317 | C | -0.0585 |
| rs442495 | 15 | 59022615 | C | -0.0599 |
| rs13316744 | 3 | 16742711 | C | -0.0549 |
| rs7068231 | 10 | 61784928 | T | -0.0579 |
| rs834398 | 5 | 160528276 | G | 0.0793 |
| rs62013908 | 16 | 5991314 | G | 0.0724 |
| rs17765431 | 14 | 70622948 | G | 0.1129 |
| rs112537817 | 12 | 62308897 | G | 0.2248 |
| rs1639034 | 7 | 2885235 | G | 0.062 |
| rs71538860 | 7 | 14051383 | G | 0.2519 |
| rs35682329 | 1 | 155121143 | G | 0.2639 |
| rs36006255 | 3 | 121770644 | C | -0.0861 |
| rs7126035 | 11 | 126381461 | C | 0.086 |
| rs76792388 | 5 | 179629485 | C | 0.0958 |
| rs59377457 | 4 | 68826930 | G | 0.0702 |
| rs17127524 | 1 | 65797988 | C | 0.1474 |
| rs181865845 | 11 | 129368087 | G | 0.1946 |
| rs11987986 | 8 | 290044 | G | 0.2104 |
| rs9567265 | 13 | 44386266 | T | 0.063 |
| rs118158255 | 11 | 81251949 | G | 0.1394 |
| rs55818489 | 17 | 43841308 | G | 0.0633 |
| rs2830477 | 21 | 28137339 | C | -0.0577 |
| rs753897 | 2 | 47102655 | G | 0.0644 |
| rs143446632 | 3 | 132738489 | C | 0.3046 |
| rs7180627 | 15 | 99067955 | A | 0.0777 |
| rs78057930 | 5 | 20541014 | G | 0.2499 |
| **rs62213385^b^** | **21** | **44158264** | **C** | **0.0729** |
| rs12449792 | 17 | 43302259 | C | -0.058 |
| rs62453586 | 7 | 5946197 | G | -0.0638 |
| rs76623552 | 13 | 113691546 | G | 0.1075 |
| rs1234820 | 12 | 48106488 | G | -0.0754 |
| rs1030985 | 8 | 73745466 | T | 0.0594 |
| rs145650600 | 14 | 90653729 | G | 0.2974 |
| rs111357651 | 13 | 109814452 | C | 0.1526 |
| rs67114353 | 4 | 181043819 | C | -0.0629 |
| rs5010942 | 1 | 42107049 | C | -0.1461 |
| rs72891514 | 18 | 37375561 | G | 0.2016 |
| rs76855300 | 15 | 63595878 | C | 0.0909 |
| rs28546867 | 2 | 1238956 | G | 0.073 |
| rs12264367 | 10 | 131320026 | G | 0.0694 |
| rs6518681 | 22 | 30609554 | A | 0.1026 |
| rs1930863 | 13 | 71000385 | T | 0.0746 |
| rs28567888 | 3 | 178431520 | C | 0.0634 |
| rs12780504 | 10 | 3788332 | G | 0.1654 |
| rs2243655 | 10 | 28344794 | T | -0.0501 |
| rs1378069 | 12 | 55170462 | C | -0.0494 |
| rs79207260 | 21 | 15616324 | C | 0.1031 |
| rs881699 | 8 | 144478004 | G | -0.0838 |
| rs12787112 | 11 | 47687147 | G | -0.0515 |
| rs72796098 | 5 | 141408367 | G | -0.1185 |
| rs72946166 | 6 | 73231438 | C | 0.1257 |
| rs78566319 | 7 | 129012006 | G | 0.1788 |
| rs79087498 | 9 | 8288563 | G | 0.1699 |
| rs80232382 | 6 | 140197462 | C | 0.108 |
| rs10991386 | 9 | 107630433 | G | 0.0567 |
| rs7581787 | 2 | 234077240 | C | 0.056 |
| ^a^Beta was taken from Mega Vascular Cognitive Impairment and Dementia (MEGAVCID) consortium. A genome-wide association meta-analysis of all-cause and vascular dementia. Alzheimers Dement. 2024;20(9):5973-5995.  ^b^This SNP was not found in the genomic data of the UK Biobank, which was then excluded in calculating the PRS for all-cause dementia. | | | | |

**Table S3** Definition and assessment of covariates

| **Covariates** | **Definition** | **Assessment** | **UK biobank**  **Data-Field ID** |
| --- | --- | --- | --- |
| Age (years) | Age in years | Difference between date of starting to wear the accelerometer and date of birth recorded by NHS | 90010, 34 |
| Sex | Men, Women | NHS derived and/or touchscreen questionnaire | 31 |
| Ethnic background | White, Non-White (Mixed, Asian, Black, Chinese, Other) | Touchscreen questionnaire: “What is your ethnic group?” | 21000 |
| Education | Higher education (college or university degree, other professional qualifications), other than higher education | Touchscreen questionnaire: “Which of the following qualifications do you have?” | 6138 |
| BMI (kg/m^2^) | Continuous | Physical examination: body mass index, calculated from height and weight measured during the initial Assessment Centre visit | 21001 |
| Townsend deprivation index | Continuous | Townsend deprivation index calculated prior to participant joining UK Biobank. Based on the preceding national census output areas. Each participant is assigned a score corresponding to the output area in  which their postcode is located. | 22189 |
| Current smoking | Yes, No | Touchscreen questionnaire: “Do you smoke tobacco now?” and “In the past, how often have you smoked tobacco?” | 20116 |
| Alcohol intake | At least once per week, less than once per week | Touchscreen questionnaire: “About how often do you drink alcohol?” | 1558 |
| Depressed mood | Yes (nearly every day or more than half the days), No (not at all or several days) | Touchscreen questionnaire: “Over the past two weeks, how often have you felt down, depressed or hopeless?” | 2050 |
| Hypertension | Yes, No | Touchscreen questionnaire and verbal interview: self-reported hypertension or anti-hypertensive medication use;  Average SBP/DBP ≥ 140/90 mmHg at baseline | 6150, 20002, 6177, 4079, 4080, 93, 94 |
| Diabetes | Yes, No | Touchscreen questionnaire and verbal interview: self-reported diabetes (diabetes, type 1 diabetes or type 2 diabetes) or medication use for lowering blood glucose;  Plasma hba1c ≥ 48 mmol/mol (6.5%) | 2443, 20002, 6153, 6177, 30750, 20003 |
| Coronary heart disease | Yes, No | Touchscreen questionnaire and verbal interview: angina, heart attack/myocardial infarction | 6150, 20002 |
| Stroke | Yes, No | Touchscreen questionnaire and verbal interview: self-reported previous stroke or transient ischaemic attack (TIA) | 6150, 20002 |
| ApoE4 carrier | Yes, No | Genomics | affy160203316  affy160203324 |

**Table S4** Movement Behaviors of 94 086 Study Participants by Baseline Characteristics

| **Characteristics** | **No. (%)** | **Sleep, h/d** | ***P*-value** | **SB, h/d** | ***P*-value** | **LIPA, h/d** | ***P*-value** | **MVPA, min/d** | ***P*-value** |
| --- | --- | --- | --- | --- | --- | --- | --- | --- | --- |
| Overall | 94 086 (100) | 8.7 (8.0-9.5) | NA | 9.4 (8.2-10.6) | NA | 4.9 (3.9-6.1) | NA | 33 (16-57) | NA |
| Age, years | | | | | | | | | |
| 40-49 | 6790 (7.2) | 8.6 (8.0-9.3) | <0.001 | 9.5 (8.2-10.7) | <0.001 | 5.0 (3.9-6.2) | <0.001 | 38 (20-62) | <0.001 |
| 50-59 | 26 469 (28.1) | 8.6 (8.0-9.4) |  | 9.5 (8.2-10.7) |  | 4.9 (3.9-6.1) |  | 36 (18-60) |  |
| 60-69 | 41 531 (44.1) | 8.8 (8.1-9.6) |  | 9.3 (8.1-10.5) |  | 5.0 (4.0-6.1) |  | 33 (16-58) |  |
| 70-79 | 19 296 (20.5) | 8.8 (8.1-9.6) |  | 9.5 (8.3-10.7) |  | 4.8 (3.8-5.9) |  | 28 (12-50) |  |
| Sex | | | | | | | | | |
| Female | 52 853 (56.2) | 8.8 (8.1-9.5) | <0.001 | 9.1 (8.0-10.3) | <0.001 | 5.3 (4.3-6.4) | <0.001 | 28 (13-49) | <0.001 |
| Male | 41 233 (43.8) | 8.7 (8.0-9.5) |  | 9.8 (8.6-11.0) |  | 4.4 (3.5-5.5) |  | 41 (21-68) |  |
| Race | | | | | | | | | |
| White^a^ | 90 868 (96.6) | 8.7 (8.1-9.5) | <0.001 | 9.4 (8.2-10.6) | 0.017 | 4.9 (3.9-6.1) | <0.001 | 33 (16-58) | <0.001 |
| Other | 3218 (3.4) | 8.5 (7.7-9.4) |  | 9.5 (8.2-10.7) |  | 5.1 (4.0-6.4) |  | 30 (14-52) |  |
| Higher educational level | | | | | | | | | |
| Yes | 55 214 (58.7) | 8.7 (8.0-9.4) | <0.001 | 9.5 (8.3-10.7) | <0.001 | 4.8 (3.8-6.0) | <0.001 | 36 (19-61) | <0.001 |
| No | 38 872 (41.3) | 8.8 (8.1-9.7) |  | 9.2 (8.0-10.5) |  | 5.1 (4.0-6.2) |  | 29 (13-51) |  |
| Townsend deprivation index | | | | | | | | | |
| Least deprived, < -3.8 | 22 994 (24.4) | 8.8 (8.1-9.5) | <0.001 | 9.3 (8.1-10.5) | <0.001 | 5.0 (4.0-6.1) | <0.001 | 33 (16-57) | <0.001 |
| -3.8 to -2.5 | 24 085 (25.6) | 8.8 (8.1-9.5) |  | 9.3 (8.2-10.5) |  | 5.0 (3.9-6.1) |  | 32 (16-57) |  |
| -2.4 to -0.2 | 23 760 (25.3) | 8.7 (8.1-9.5) |  | 9.4 (8.2-10.6) |  | 5.0 (3.9-6.1) |  | 33 (16-57) |  |
| Most deprived, > -0.2 | 23 247 (24.7) | 8.7 (8.0-9.5) |  | 9.6 (8.3-10.8) |  | 4.8 (3.8-6.0) |  | 34 (17-59) |  |
| BMI, kg/m^2^ | | | | | | | | | |
| <18.5 kg/m^2^ | 497 (0.5) | 8.6 (8.0-9.4) | 0.022 | 8.6 (7.5-9.9) | <0.001 | 5.7 (4.4-6.9) | <0.001 | 42 (23-68) | <0.001 |
| 18.5-24.9 kg/m^2^ | 36 061 (38.3) | 8.7 (8.1-9.5) |  | 9.0 (7.9-10.2) |  | 5.2 (4.2-6.4) |  | 39 (21-64) |  |
| 25.0-29.9 kg/m^2^ | 39 107 (41.6) | 8.7 (8.0-9.5) |  | 9.5 (8.3-10.7) |  | 4.8 (3.8-6.0) |  | 33 (17-57) |  |
| >29.9 kg/m^2^ | 18 421 (19.6) | 8.7 (8.0-9.6) |  | 10.0 (8.8-11.2) |  | 4.5 (3.5-5.6) |  | 21 (9-41) |  |
| Current smoker | | | | | | | | | |
| Yes | 6493 (6.9) | 8.8 (8.0-9.6) | 0.010 | 9.6 (8.3-10.9) | <0.001 | 4.7 (3.7-6.0) | <0.001 | 27 (11-50) | <0.001 |
| No | 87 593 (93.1) | 8.7 (8.0-9.5) |  | 9.4 (8.2-10.6) |  | 4.9 (3.9-6.1) |  | 34 (17-58) |  |
| Alcohol intake | | | | | | | | | |
| ≥1 time per week | 69 595 (74.0) | 8.7 (8.1-9.5) | <0.001 | 9.4 (8.2-10.6) | 0.013 | 4.9 (3.9-6.0) | <0.001 | 36 (18-60) | <0.001 |
| <1 time per week | 24 491 (26.0) | 8.8 (8.0-9.6) |  | 9.4 (8.1-10.6) |  | 5.0 (3.9-6.2) |  | 27 (12-49) |  |
| Depressed mood | | | | | | | | | |
| Yes | 3032 (3.2) | 8.8 (8.1-9.7) | <0.001 | 9.4 (8.2-10.8) | 0.045 | 4.8 (3.7-6.0) | <0.001 | 26 (10-49) | <0.001 |
| No | 91 054 (96.8) | 8.7 (8.0-9.5) |  | 9.4 (8.2-10.6) |  | 4.9 (3.9-6.1) |  | 33 (16-58) |  |
| Diabetes | | | | | | | | | |
| Yes | 3781 (4.0) | 8.8 (8.0-9.7) | 0.002 | 10.1 (8.8-11.4) | <0.001 | 4.3 (3.3-5.5) | <0.001 | 20 (7-41) | <0.001 |
| No | 90 305 (96.0) | 8.7 (8.1-9.5) |  | 9.4 (8.2-10.6) |  | 5.0 (3.9-6.1) |  | 34 (17-58) |  |
| Hypertension | | | | | | | | | |
| Yes | 47 442 (50.4) | 8.8 (8.1-9.6) | <0.001 | 9.5 (8.3-10.7) | <0.001 | 4.8 (3.8-5.9) | <0.001 | 31 (14-55) | <0.001 |
| No | 46 644 (49.6) | 8.7 (8.0-9.4) |  | 9.3 (8.1-10.5) |  | 5.1 (4.0-6.2) |  | 36 (18-60) |  |
| Coronary heart disease | | | | | | | | | |
| Yes | 2940 (3.1) | 8.9 (8.1-9.8) | <0.001 | 9.8 (8.6-11.1) | <0.001 | 4.3 (3.4-5.5) | <0.001 | 24 (10-46) | <0.001 |
| No | 91 146 (96.9) | 8.7 (8.0-9.5) |  | 9.4 (8.2-10.6) |  | 4.9 (3.9-6.1) |  | 33 (16-58) |  |
| Stroke | | | | | | | | | |
| Yes | 1060 (1.1) | 8.8 (8.0-9.7) | 0.117 | 9.8 (8.6-11.1) | <0.001 | 4.5 (3.5-5.7) | <0.001 | 24 (10-47) | <0.001 |
| No | 93 026 (98.9) | 8.7 (8.0-9.5) |  | 9.4 (8.2-10.6) |  | 4.9 (3.9-6.1) |  | 33 (16-58) |  |
| ApoE4 carrier | | | | | | | | | |
| Yes | 22 528 (23.9) | 8.7 (8.0-9.5) | <0.001 | 9.4 (8.1-10.6) | 0.020 | 5.0 (3.9-6.1) | <0.001 | 34 (17-58) | <0.001 |
| No | 71 558 (76.1) | 8.7 (8.1-9.5) |  | 9.4 (8.2-10.6) |  | 4.9 (3.9-6.1) |  | 33 (16-57) |  |
| Abbreviations: BMI, body mass index; LIPA, light-intensity physical activity; MVPA, moderate-to-vigorous physical activity; NA, not applicable; SB, sedentary behavior.  ^a^White included British, Irish, and any other white background.  ^b^Other was defined as Asian, Black, Chinese, Mixed, or other ethnic background. | | | | | | | | | |

| **Table S5** Associations of reallocation of time between different types of movement behaviors and incident dementia: competing risk model (n=94 086) | | | | |
| --- | --- | --- | --- | --- |
|  | **Sleep** | **SB** | **LIPA** | **MVPA** |
| Replace **sleep** with | Replaced | 1.10 (1.03, 1.17) | 1.02 (0.95, 1.09) | 0.83 (0.71, 0.98) |
| Replace **SB** with | 0.91 (0.86, 0.97) | Replaced | 0.93 (0.88, 0.98) | 0.76 (0.65, 0.88) |
| Replace **LIPA** with | 0.98 (0.91, 1.06) | 1.08 (1.02, 1.14) | Replaced | 0.82 (0.69, 0.96) |
| Replace **MVPA** with | 1.20 (1.02, 1.42) | 1.32 (1.13, 1.54) | 1.22 (1.04, 1.44) | Replaced |
| Abbreviations: SB, sedentary behavior; LIPA, light-intensity physical activity; MVPA, moderate-to-vigorous physical activity.  Adjusted covariates: age, sex, race, education, body mass index, Townsend deprivation index, current smoking, current alcohol consumption, depressed mood, hypertension, diabetes, coronary heart disease, stroke, apolipoprotein E4 status. | | | | |

| **Table S6** Associations of reallocation of time between different types of movement behaviors and incident dementia after excluding dementia diagnosed within 2 years since baseline (n=94 037) | | | | |
| --- | --- | --- | --- | --- |
|  | **Sleep** | **SB** | **LIPA** | **MVPA** |
| Replace **sleep** with | Replaced | 1.13 (1.06, 1.21) | 1.03 (0.95, 1.11) | 0.83 (0.70, 0.98) |
| Replace **SB** with | 0.89 (0.83, 0.95) | Replaced | 0.91 (0.86, 0.96) | 0.73 (0.63, 0.86) |
| Replace **LIPA** with | 0.97 (0.90, 1.05) | 1.10 (1.04, 1.17) | Replaced | 0.81 (0.68, 0.95) |
| Replace **MVPA** with | 1.21 (1.02, 1.43) | 1.37 (1.17, 1.60) | 1.24 (1.05, 1.47) | Replaced |
| Abbreviations: SB, sedentary behavior; LIPA, light-intensity physical activity; MVPA, moderate-to-vigorous physical activity.  Adjusted covariates: age, sex, race, education, body mass index, Townsend deprivation index, current smoking, current alcohol consumption, depressed mood, hypertension, diabetes, coronary heart disease, stroke, apolipoprotein E4 status. | | | | |

| **Table S7** Associations of reallocation of time between different types of movement behaviors and mortality after excluding death within 2 years since baseline (n=95 748) | | | | |
| --- | --- | --- | --- | --- |
|  | **Sleep** | **SB** | **LIPA** | **MVPA** |
| Replace **sleep** with | Replaced | 1.02 (0.99, 1.05) | 0.93 (0.90, 0.96) | 0.73 (0.67, 0.79) |
| Replace **SB** with | 0.98 (0.95, 1.01) | Replaced | 0.91 (0.89, 0.93) | 0.71 (0.66, 0.77) |
| Replace **LIPA** with | 1.08 (1.04, 1.11) | 1.10 (1.07, 1.13) | Replaced | 0.78 (0.72, 0.85) |
| Replace **MVPA** with | 1.38 (1.27, 1.49) | 1.41 (1.30, 1.52) | 1.28 (1.18, 1.39) | Replaced |
| Abbreviations: SB, sedentary behavior; LIPA, light-intensity physical activity; MVPA, moderate-to-vigorous physical activity.  Adjusted covariates: age, sex, race, education, body mass index, Townsend deprivation index, current smoking, current alcohol consumption, depressed mood, hypertension, diabetes, coronary heart disease, stroke, apolipoprotein E4 status. | | | | |

| **Table S8** Associations of reallocation of time between different types of movement behaviors and incident dementia after excluding participants aged <50 years at baseline (n=87 296) | | | | |
| --- | --- | --- | --- | --- |
|  | **Sleep** | **SB** | **LIPA** | **MVPA** |
| Replace **sleep** with | Replaced | 1.10 (1.03, 1.18) | 1.01 (0.94, 1.09) | 0.83 (0.70, 0.97) |
| Replace **SB** with | 0.91 (0.85, 0.97) | Replaced | 0.92 (0.87, 0.97) | 0.75 (0.64, 0.87) |
| Replace **LIPA** with | 0.99 (0.92, 1.07) | 1.09 (1.03, 1.15) | Replaced | 0.82 (0.69, 0.96) |
| Replace **MVPA** with | 1.21 (1.03, 1.43) | 1.34 (1.14, 1.56) | 1.23 (1.04, 1.45) | Replaced |
| Abbreviations: SB, sedentary behavior; LIPA, light-intensity physical activity; MVPA, moderate-to-vigorous physical activity.  Adjusted covariates: age, sex, race, education, body mass index, Townsend deprivation index, current smoking, current alcohol consumption, depressed mood, hypertension, diabetes, coronary heart disease, stroke, apolipoprotein E4 status. | | | | |

| **Table S9** Associations of reallocation of time between different types of movement behaviors and incident dementia among participants with high or low duration of sedentary behavior (n=94 086) | | | | |
| --- | --- | --- | --- | --- |
|  | **Sleep** | **SB** | **LIPA** | **MVPA** |
| **High SB duration** (n=31 359)^*^ | | | | |
| Replace **sleep** with | Replaced | 1.36 (1.23, 1.50) | 1.03 (0.91, 1.17) | 1.01 (0.75, 1.37) |
| Replace **SB** with | 0.74 (0.67, 0.82) | Replaced | 0.76 (0.68, 0.85) | 0.75 (0.56, 1.00) |
| Replace **LIPA** with | 0.97 (0.86, 1.10) | 1.32 (1.17, 1.48) | Replaced | 0.98 (0.72, 1.34) |
| Replace **MVPA** with | 0.99 (0.73, 1.34) | 1.34 (1.00, 1.80) | 1.02 (0.74, 1.39) | Replaced |
| **Low SB duration** (n=62 727)^*^ | | | | |
| Replace **sleep** with | Replaced | 0.93 (0.85, 1.02) | 0.98 (0.90, 1.06) | 0.74 (0.61, 0.89) |
| Replace **SB** with | 1.07 (0.98, 1.18) | Replaced | 1.05 (0.96, 1.15) | 0.79 (0.65, 0.96) |
| Replace **LIPA** with | 1.02 (0.94, 1.11) | 0.95 (0.87, 1.04) | Replaced | 0.75 (0.62, 0.92) |
| Replace **MVPA** with | 1.36 (1.12, 1.64) | 1.26 (1.04, 1.54) | 1.33 (1.09, 1.61) | Replaced |
| ^*^High SB duration was defined as the top tertile of SB duration, while low SB duration was defined as the bottom two tertiles of SB duration.  Abbreviations: SB, sedentary behavior; LIPA, light-intensity physical activity; MVPA, moderate-to-vigorous physical activity.  Adjusted covariates: age, sex, race, education, body mass index, Townsend deprivation index, current smoking, current alcohol consumption, depressed mood, hypertension, diabetes, coronary heart disease, stroke, apolipoprotein E4 status. | | | | |

| **Table S10** Associations of reallocation of time between different types of movement behaviors and mortality among participants with high or low duration of sedentary behavior (n=96 295) | | | | |
| --- | --- | --- | --- | --- |
|  | **Sleep** | **SB** | **LIPA** | **MVPA** |
| **High SB duration** (n=32 113)^*^ | | | | |
| Replace **sleep** with | Replaced | 1.12 (1.07, 1.18) | 0.88 (0.83, 0.93) | 0.59 (0.51, 0.69) |
| Replace **SB** with | 0.89 (0.85, 0.94) | Replaced | 0.78 (0.74, 0.83) | 0.53 (0.45, 0.61) |
| Replace **LIPA** with | 1.14 (1.08, 1.20) | 1.28 (1.21, 1.34) | Replaced | 0.67 (0.57, 0.79) |
| Replace **MVPA** with | 1.70 (1.46, 1.97) | 1.90 (1.64, 2.21) | 1.49 (1.27, 1.75) | Replaced |
| **Low SB duration** (n=64 182)^*^ | | | | |
| Replace **sleep** with | Replaced | 0.94 (0.90, 0.98) | 0.92 (0.89, 0.95) | 0.76 (0.69, 0.82) |
| Replace **SB** with | 1.06 (1.02, 1.11) | Replaced | 0.98 (0.94, 1.01) | 0.80 (0.74, 0.88) |
| Replace **LIPA** with | 1.09 (1.05, 1.13) | 1.03 (0.99, 1.07) | Replaced | 0.82 (0.76, 0.90) |
| Replace **MVPA** with | 1.32 (1.22, 1.44) | 1.25 (1.14, 1.36) | 1.21 (1.11, 1.32) | Replaced |
| ^*^High SB duration was defined as the top tertile of SB duration, while low SB duration was defined as the bottom two tertiles of SB duration.  Abbreviations: SB, sedentary behavior; LIPA, light-intensity physical activity; MVPA, moderate-to-vigorous physical activity.  Adjusted covariates: age, sex, race, education, body mass index, Townsend deprivation index, current smoking, current alcohol consumption, depressed mood, hypertension, diabetes, coronary heart disease, stroke, apolipoprotein E4 status. | | | | |

| **Table S11** Associations of reallocation of time between different types of movement behaviors and premature death among participants with high or low duration of sedentary behavior (n=96 295) | | | | |
| --- | --- | --- | --- | --- |
|  | **Sleep** | **SB** | **LIPA** | **MVPA** |
| **High SB duration** (n=32 113)^*^ | | | | |
| Replace **sleep** with | Replaced | 1.12 (1.07, 1.18) | 0.88 (0.83, 0.93) | 0.60 (0.51, 0.70) |
| Replace **SB** with | 0.89 (0.85, 0.94) | Replaced | 0.78 (0.74, 0.83) | 0.53 (0.46, 0.62) |
| Replace **LIPA** with | 1.14 (1.07, 1.20) | 1.28 (1.21, 1.35) | Replaced | 0.68 (0.58, 0.80) |
| Replace **MVPA** with | 1.68 (1.43, 1.96) | 1.88 (1.62, 2.19) | 1.48 (1.25, 1.74) | Replaced |
| **Low SB duration** (n=64 182)^*^ | | | | |
| Replace **sleep** with | Replaced | 0.94 (0.90, 0.98) | 0.92 (0.88, 0.95) | 0.76 (0.70, 0.83) |
| Replace **SB** with | 1.07 (1.02, 1.11) | Replaced | 0.98 (0.94, 1.02) | 0.81 (0.74, 0.89) |
| Replace **LIPA** with | 1.09 (1.05, 1.13) | 1.02 (0.98, 1.07) | Replaced | 0.83 (0.76, 0.91) |
| Replace **MVPA** with | 1.32 (1.20, 1.44) | 1.23 (1.12, 1.35) | 1.20 (1.10, 1.32) | Replaced |
| ^*^High SB duration was defined as the top tertile of SB duration, while low SB duration was defined as the bottom two tertiles of SB duration.  Abbreviations: SB, sedentary behavior; LIPA, light-intensity physical activity; MVPA, moderate-to-vigorous physical activity.  Adjusted covariates: age, sex, race, education, body mass index, Townsend deprivation index, current smoking, current alcohol consumption, depressed mood, hypertension, diabetes, coronary heart disease, stroke, apolipoprotein E4 status. | | | | |

| **Table S12** Associations of reallocation of time between different types of movement behaviors and dementia, stratified by baseline age (n=94 086) | | | |
| --- | --- | --- | --- |
|  | Age <60 years (n=33 259) | Age ≥60 years (n=60 827) | P for interaction |
| Replace **sleep** with **SB** | 0.90 (0.64, 1.26) | 1.11 (1.04, 1.19) | 0.234 |
| Replace **sleep** with **LIPA** | 0.93 (0.66, 1.31) | 1.01 (0.94, 1.09) | 0.645 |
| Replace **sleep** with **MVPA** | 0.85 (0.45, 1.60) | 0.81 (0.69, 0.96) | 0.885 |
| Replace **SB** with **sleep** | 1.11 (0.79, 1.56) | 0.90 (0.84, 0.96) | 0.236 |
| Replace **SB** with **LIPA** | 1.03 (0.82, 1.30) | 0.91 (0.86, 0.97) | 0.308 |
| Replace **SB** with **MVPA** | 0.95 (0.53, 1.68) | 0.73 (0.63, 0.86) | 0.049 |
| Replace **LIPA** with **sleep** | 1.08 (0.76, 1.52) | 0.99 (0.92, 1.06) | 0.630 |
| Replace **LIPA** with **SB** | 0.97 (0.77, 1.22) | 1.10 (1.03, 1.16) | 0.300 |
| Replace **LIPA** with **MVPA** | 0.92 (0.49, 1.72) | 0.80 (0.68, 0.95) | 0.673 |
| Replace **MVPA** with **sleep** | 1.17 (0.63, 2.21) | 1.23 (1.04, 1.45) | 0.880 |
| Replace **MVPA** with **SB** | 1.06 (0.59, 1.88) | 1.36 (1.16, 1.60) | 0.417 |
| Replace **MVPA** with **LIPA** | 1.09 (0.58, 2.04) | 1.25 (1.05, 1.48) | 0.681 |
| Abbreviations: SB, sedentary behavior; LIPA, light-intensity physical activity; MVPA, moderate-to-vigorous physical activity.  Adjusted covariates: age, sex, race, education, body mass index, Townsend deprivation index, current smoking, current alcohol consumption, depressed mood, hypertension, diabetes, coronary heart disease, stroke, apolipoprotein E4 status. | | | |

| **Table S13** Associations of reallocation of time between different types of movement behaviors and mortality, stratified by baseline age (n=96 295) | | | |
| --- | --- | --- | --- |
|  | Age <60 years (n=34 048) | Age ≥60 years (n=62 247) | P for interaction |
| Replace **sleep** with **SB** | 0.97 (0.89, 1.05) | 1.02 (1.00, 1.05) | 0.253 |
| Replace **sleep** with **LIPA** | 0.86 (0.78, 0.94) | 0.92 (0.89, 0.95) | 0.181 |
| Replace **sleep** with **MVPA** | 0.78 (0.64, 0.95) | 0.70 (0.64, 0.76) | 0.325 |
| Replace **SB** with **sleep** | 1.04 (0.95, 1.12) | 0.98 (0.95, 1.00) | 0.177 |
| Replace **SB** with **LIPA** | 0.89 (0.83, 0.95) | 0.90 (0.87, 0.92) | 0.764 |
| Replace **SB** with **MVPA** | 0.80 (0.67, 0.97) | 0.68 (0.63, 0.74) | 0.114 |
| Replace **LIPA** with **sleep** | 1.17 (1.06, 1.28) | 1.09 (1.06, 1.13) | 0.163 |
| Replace **LIPA** with **SB** | 1.13 (1.06, 1.21) | 1.12 (1.09, 1.15) | 0.807 |
| Replace **LIPA** with **MVPA** | 0.91 (0.74, 1.10) | 0.76 (0.70, 0.83) | 0.102 |
| Replace **MVPA** with **sleep** | 1.29 (1.06, 1.57) | 1.43 (1.32, 1.55) | 0.229 |
| Replace **MVPA** with **SB** | 1.25 (1.04, 1.50) | 1.47 (1.35, 1.59) | 0.113 |
| Replace **MVPA** with **LIPA** | 1.10 (0.91, 1.35) | 1.31 (1.21, 1.43) | 0.110 |
| Abbreviations: SB, sedentary behavior; LIPA, light-intensity physical activity; MVPA, moderate-to-vigorous physical activity.  Adjusted covariates: age, sex, race, education, body mass index, Townsend deprivation index, current smoking, current alcohol consumption, depressed mood, hypertension, diabetes, coronary heart disease, stroke, apolipoprotein E4 status. | | | |

| **Table S14** Associations of reallocation of time between different types of movement behaviors and premature death, stratified by baseline age (n=96 295) | | | |
| --- | --- | --- | --- |
|  | Age <60 years (n=34 048) | Age ≥60 years (n=62 247) | P for interaction |
| Replace **sleep** with **SB** | 0.97 (0.89, 1.05) | 1.03 (1.00, 1.06) | 0.180 |
| Replace **sleep** with **LIPA** | 0.86 (0.78, 0.94) | 0.92 (0.89, 0.95) | 0.181 |
| Replace **sleep** with **MVPA** | 0.78 (0.64, 0.95) | 0.70 (0.64, 0.76) | 0.325 |
| Replace **SB** with **sleep** | 1.04 (0.95, 1.12) | 0.97 (0.94, 1.00) | 0.120 |
| Replace **SB** with **LIPA** | 0.89 (0.83, 0.95) | 0.89 (0.87, 0.91) | 1.000 |
| Replace **SB** with **MVPA** | 0.80 (0.67, 0.97) | 0.68 (0.63, 0.74) | 0.114 |
| Replace **LIPA** with **sleep** | 1.17 (1.06, 1.28) | 1.09 (1.06, 1.13) | 0.163 |
| Replace **LIPA** with **SB** | 1.13 (1.06, 1.21) | 1.12 (1.10, 1.16) | 0.807 |
| Replace **LIPA** with **MVPA** | 0.91 (0.74, 1.10) | 0.76 (0.70, 0.84) | 0.106 |
| Replace **MVPA** with **sleep** | 1.29 (1.06, 1.57) | 1.43 (1.31, 1.56) | 0.348 |
| Replace **MVPA** with **SB** | 1.25 (1.04, 1.50) | 1.47 (1.35, 1.60) | 0.116 |
| Replace **MVPA** with **LIPA** | 1.10 (0.91, 1.35) | 1.31 (1.20, 1.43) | 0.113 |
| Abbreviations: SB, sedentary behavior; LIPA, light-intensity physical activity; MVPA, moderate-to-vigorous physical activity.  Adjusted covariates: age, sex, race, education, body mass index, Townsend deprivation index, current smoking, current alcohol consumption, depressed mood, hypertension, diabetes, coronary heart disease, stroke, apolipoprotein E4 status. | | | |

| **Table S15** Comparison between baseline characteristics between participants included (n=94 086) and excluded (n=9493) due to invalid accelerometer and genomic data, history of dementia, or missing data of covariates | | | |
| --- | --- | --- | --- |
| **Characteristics** | **Participants included (n=94 086)** | **Participants excluded (n=9493)** | ***P* value^a^** |
| Age, mean (SD), y | 62.3 (7.8) | 61.2 (8.0) | <.001 |
| Female | 52 853 (56.2) | 5375 (56.6) | .40 |
| White | 90 868 (96.6) | 8969 (94.5) | <.001 |
| Higher education | 55 214 (58.7) | 5409 (57.0) | .001 |
| Townsend deprivation index, median (IQR) | -2.5 (3.6) | -2.2 (3.9) | <.001 |
| BMI, mean (SD) | 26.7 (4.5) | 27.0 (4.8) | <.001 |
| Time spent in sleep, median (IQR) | 8.7 (1.5) | 10.0 (12.1) | <.001 |
| Time spent in SB, median (IQR) | 9.4 (2.4) | 8.0 (7.5) | <.001 |
| Time spent in LIPA, median (IQR) | 4.9 (2.2) | 3.2 (5.5) | <.001 |
| Time spent in MVPA, median (IQR) | 33 (41) | 9 (40) | <.001 |
| Current smoking | 6493 (6.9) | 772 (8.1) | <.001 |
| Current drinking | 69 595 (74.0) | 7027 (74.0) | .91 |
| Depressed mood | 3032 (3.2) | 353 (3.7) | .01 |
| Diabetes | 3781 (4.0) | 397 (4.2) | .44 |
| Hypertension | 47 442 (50.4) | 4552 (48.0) | <.001 |
| Coronary heart disease | 2940 (3.1) | 276 (2.9) | .24 |
| Stroke | 1060 (1.1) | 112 (1.2) | .64 |
| ApoE4 carrier | 22 528 (23.9) | 1748 (18.4) | <.001 |
| Abbreviations: SD, standard deviation; IQR, interquartile range; BMI, body mass index; SB, sedentary behavior; LIPA, light-intensity physical activity; MVPA, moderate-to-vigorous physical activity.  The results are presented as mean (SD), median (IQR), or No. (%).  ^a^Calculated by using a *t* test, Mann–Whitney U test, or chi-square test. | | | |
